# MaMYB12 and MaMYB308 antagonistically regulate flavonoid biosynthesis in mulberry (*Morus alba*): Implications for functional food ingredient development

**DOI:** 10.1016/j.fochms.2026.100395

**Published:** 2026-03-24

**Authors:** Shengmei Han, Teng Zhao, Jiaqi Zhao, Xiaoyan Liu, Jing Xiao, Hong Huang, Zhaoyang Liu, Yingping Gai, Xianling Ji

**Affiliations:** a College of Forestry, Shandong Agricultural University, Taian, Shandong 271018, People's Republic of China; b College of Life Science, Shandong Agricultural University, Taian, Shandong 271018, People's Republic of China.

**Keywords:** Mulberry, R2R3-MYB transcription factor, Flavonoid biosynthesis, Functional food, Bioactive ingredients

## Abstract

Mulberry (*Morus alba*), a tree with multipurpose applications, is a promising source of bioactive flavonoids for functional foods. Mulberry-derived flavonoids possess prominent antioxidant, anti-inflammatory, and cardioprotective activities, making them valuable functional ingredients for nutraceutical products. Elucidating the transcriptional regulation of flavonoid biosynthesis is essential for targeted molecular breeding to enhance flavonoid accumulation in mulberry, thereby supporting sustainable production of high-value food ingredients. In this study, five R2R3-MYB transcription factors whose expression correlates with flavonoid accumulation during mulberry fruit development were identified. Among these, MaMYB12 (subgroup 7, SG7) functions as a transcriptional activator. It transient overexpression in mulberry leaves significantly elevated the content of 30 flavonoid metabolites by directly activating the promoters of key flavonoid biosynthetic genes. In contrast, MaMYB308 (SG4) functions as a strong repressor, reducing the accumulation of 106 flavonoid metabolites through directly suppressing of key flavonoid biosynthetic genes. The direct binding of both MaMYB12 and MaMYB308 to target gene promoters was verified by yeast one-hybrid assays and electrophoretic mobility shift assays. Their antagonistic regulatory roles were further confirmed in stably transformed mulberry hairy roots. Additionally, extracts from *MaMYB12*-overexpressing hairy roots exhibited significantly enhanced antioxidant activity, directly linking this genetic manipulation to improved functional food properties. Our findings uncover a key transcriptional module governing flavonoid metabolism in mulberry and present practical genetic tools for the molecular breeding of mulberry varieties with optimized flavonoid profiles, advancing their utility as functional food ingredients.

## Highlights

• MaMYB12 and MaMYB308 function as antagonistic regulators of flavonoid biosynthesis in mulberry.
• MaMYB12 activates 30 bioactive flavonoids via targeting Ma4CL/MaF3H/MaFLS.
• MaMYB308 broadly represses 106 flavonoids across subclasses by suppressing Ma4CL/MaCHS/MaFLS/MaANS.
• Genetic targets and breeding tools support breeding flavonoid‑rich mulberry for functional foods.

## 1 Introduction

Functional foods rich in natural bioactive compounds have attracted extensive consumer attention in recent years due to their potential health-promoting effects (Essa et al., 2023). Flavonoids, as a major category of plant secondary metabolites, are highly sought-after in functional food development because of their diverse biological activities such as antioxidant, anti-inflammatory, and cardioprotective effects (Jomova et al., 2025; Ma et al., 2024; Wang & Chen, 2025). The rising market demand for plant-sourced flavonoids has driven extensive research on exploring sustainable raw material sources and optimizing flavoniod production through metabolic engineering (Shen et al., 2022; Wen et al., 2021).

Mulberry (*Morus alba*), traditionally cultivated for sericulture, has emerged as a promising candidate for the production of functional food bioactive ingredient in recent years (Maqsood et al., 2022). Its various parts, including leaves, fruits, and bark, are abundant in bioactive flavonoids, including morin, quercetin derivatives, and glabranin, all of which have been proven to exert significant health-beneficial effects (Hu et al., 2022; Ma et al., 2022; Uyak et al., 2024; Xue et al., 2025). Products derived from mulberry, like leaf tea, fruit juice, and extracts, are becoming increasingly popular as functional foods, emphasizing the need for targeted breeding approaches to both increase flavonoid content and fine-tune their composition.

The biosynthetic route to flavonoids is largely evolutionarily conserved, originates from the core phenylpropanoid metabolic pathway (Wen et al., 2020). The carbon skeleton of flavonoids is constructed through a series of sequential enzymatic reactions. Starting with phenylalanine, phenylalanine ammonia-lyase (PAL), cinnamate-4-hydroxylase (C4H), and 4-coumaroyl-CoA ligase (4CL) catalyze the formation of coumaroyl-CoA, a common precursor for all flavonoids. This precursor then enters the flavonoid-specific pathway under the catalysis of chalcone synthase (CHS), followed by the actions of chalcone isomerase (CHI) and flavanone-3-hydroxylase (F3H), and finally diverges into different flavonoid subclasses including flavones, flavonols, anthocyanins, and proanthocyanidins (Liu et al., 2021; Zhuang et al., 2023; Ma et al., 2024). Other critical enzymes involved in this pathway include flavonol synthase (FLS), dihydroflavonol 4-reductase (DFR), anthocyanidin synthase (ANS), and anthocyanidin 3-O-glucosyltransferase (3GT). The spatiotemporal-specific expression of these structural genes determines the accumulation site, timing, and composition of flavonoids in plants, and this process is tightly governed at the transcriptional level (Zhang et al., 2025).

The transcriptional regulation of the flavonoid pathway involves a complex network, in which R2R3-MYB transcription factors play a central role as master regulators (Qin et al., 2023; Xu et al., 2021). In *Arabidopsis*, R2R3-MYBs involved in flavonoid biosynthesis are mainly categorized within subgroups SG4 and SG7 (Czemmel et al., 2009; Feng et al., 2004; Gonzalez et al., 2008; Shi et al., 2024; Stracke et al., 2001; Stracke et al., 2007; Wu et al., 2022). Generally, SG7 members function as activators, whereas most SG4 members (e.g., AtMYB4, VvMYBC2-L2) are repressors, which inhibits the expression of target genes to maintain the metabolic homeostasis of flavonoids in plants (Jin et al., 2000; Xie et al., 2019; Yan et al., 2021; Yoshida et al., 2015). The dynamic balance between transcriptional activators and repressors enables plants to flexibly adjust the biosynthesis of specialized metabolites in response to internal and external signals. However, the specific transcriptional mechanisms governing flavonoid biosynthesis in mulberry remain largely unexplored, particularly compared with well-studied herbaceous model plants. This knowledge gap has become a major bottleneck for the development of precision breeding strategies to improve flavonoid yields for mulberry-based functional food applications.

Therefore, the present study aimed to systematically identify R2R3-MYB transcription factors that regulate flavonoid biosynthesis in mulberry, functionally characterize the key activator and repressor among them, elucidate their underlying molecular regulatory mechanisms, and validate their antagonistic regulatory relationship. The novelty of this research lies in the identification of a pair of antagonistic R2R3-MYBs (MaMYB12 and MaMYB308) that directly regulate distinct key structural genes to modulate flavonoid metabolism in mulberry, a woody plant with important functional food value. Our study provides insights into the molecular mechanisms regulating flavonoid metabolism in mulberry, offering a genetic module for precision breeding to enhance the production of high-value functional food ingredients.

## 2 Materials and methods

### 2.1 Plant materials

The mulberry varieties ‘Guisang You 62’ and ‘Da Shi’, as well as tobacco (*Nicotiana benthamiana*), were cultivated in controlled-environment growth chambers. Conditions were set to a 16 h light/8 h dark photoperiod, 60%–70% relative humidity, and temperatures of 25 °C for mulberry and 22 °C for tobacco.

### 2.2 Determination of flavonoid content

Total flavonoid content was measured using the aluminum nitrate‑sodium nitrite colorimetric method (Bouyahya et al., 2016). Briefly, 0.1 g of dried leaf powder was extracted with 70% (*v*/v) ethanol by ultrasonication at 25 °C for 30 min (40 kHz, 500 W). The obtained extract was reacted with sodium nitrite and aluminum nitrate reagents for colour reaction, and the absorbance was measured at 510 nm. The quantification of total flavonoids was performed based on a standard curve established with rutin as the reference standard.

### 2.3 Identification, cloning, and sequence analysis of MYB genes

Candidate MYB genes associated with flavonoid biosynthesis were identified by correlating their expression profiles from a mulberry fruit transcriptome database (fruits collected at three developmental stages: MG (mature green, 20 days after flowering), MR (mature red, 30 days after flowering), and MP (mature purple, 40 days after flowering), with the observed patterns of flavonoid accumulation. Total RNA was extracted from young mulberry leaves using TRIzol reagent (Invitrogen Carlsbad, CA, USA), and treated with DNase I. cDNA was synthesized using the RevertAid First Strand cDNA Synthesis Kit (Invitrogen Carlsbad, CA, USA). Primers specific to each gene were designed based on the *M. notabilis* genome sequence, and the full-length coding sequences were amplified by PCR. The PCR products were cloned into the pEASY-Blunt Zero vector (TransGen Biotech, China), and confirmed by sequencing. Phylogenetic relationships were analyzed using MEGA 6.0 with the neighbor-joining method, and conserved motif analysis of MYB proteins was performed using DNAMAN software.

### 2.4 Vector construction and transient infection of mulberry leaves

The coding sequences (CDS) of target genes were inserted into the PNGAL plant expression vector through homologous recombination. The resulting plasmids were transformed into *Agrobacterium tumefaciens* GV3101 by the heat shock method. Bacterial cultures were pelleted and resuspended in infiltration buffer (10 mmol·L^−1^ MES, 10 m mol·L^−1^ MgCl_2_, 200 μmol·L^−1^ AS, pH 5.7) to an OD_600_ of 0.8, and then incubated at room temperature for 3 h. For infiltration, mulberry leaves were submerged in the bacterial suspension and subjected to a vacuum (0.08 MPa) for 15 min. Leaf samples were collected for analysis 3–5 d post-infiltration.

### 2.5 Targeted metabolomics analysis

Targeted metabolomics analysis of flavonoids was performed using an ultra-performance liquid chromatography-tandem mass spectrometry (UPLC-MS/MS) system (UPLC, SHIMADZU Nexera X2; MS, Applied Biosystems 4500 Q TRAP). Samples were flash-frozen, freeze-dried, and ground to a fine powder. Metabolites were extracted with 70% (*v*/v) methanol containing internal standards (d6-naringenin,1 μg·mL^−1^ final concentration) by ultrasonication at 25 °C for 20 min. The extract was centrifugated at 12, 000 ×g for 10 min at 4 °C, and the supernatant was filtered through a 0.22 μm organic filter membrane, supernatants were analyzed using an Agilent SB-C18 column (1.8 μm, 2.1 × 100 mm) using a mobile phase gradient of 0.1% formic acid in water and acetonitrile. Mass spectrometric detection was carried out on a QTRAP 4500 system in multiple reaction monitoring (MRM) mode. The operating parameters of the ESI source were set as follows: turbo spray ion source; source temperature, 550 °C; ion spray voltage 5500 V (positive)/−4500 V (negative); ion source gas I (GSI), gas II(GSII), curtain gas (CUR) were set at 50, 60, and 25 psi, respectively; collision-activated dissociation (CAD) was set to high. Instrument tuning and mass calibration were performed using polypropylene glycol solutions. Flavonoids were identified by matching their retention times and characteristic ion pairs with a self-built database (Lian Chuan Biotechnology Co., Ltd.), which was validated with more than 300 authentic standards for major flavonoid classes. Differential accumulated metabolites were defined as those with a fold change ≥2.0 or ≤ 0.5 and *p* < 0.05.

### 2.6 RNA extraction and real-time quantitative PCR (qRT-PCR)

Total RNA was isolated using a Plant Total RNA Extraction Kit, followed by DNase I treatment. cDNA synthesis was performed with the PrimeScript™ RT reagent Kit (TaKaRa Bio, Dalian, China). qRT-PCR was conducted on a Bio-Rad CFX96 real-time PCR system using SYBR® Premix Ex Taq™ II (TaKaRa Bio, Dalian, China). The mulberry *Actin* gene (GenBank: DQ785808.1) was used as an endogenous control for normalization. Relative gene expression levels were calculated using the 2^-ΔΔCt^ method (Livak & Schmittgen, 2001). All qRT-PCR experiments were performed with three biological replicates, and each biological replicate included three technical replicates.

### 2.7 Promoter activity assays

For GUS reporter assay, the promoter regions (approximately 2000 bp upstream of the start codon) of candidate genes were amplified and cloned into the pCAMBIA1301 vector (TransGen Biotech, China), which was inserted upstream of the *GUS* reporter gene. The recombinant constructs were transiently expressed in tobacco leaves via *Agrobacterium*-mediated infiltration. After 48 h of cultivation, leaves were stained with X-Gluc solution (1 mg·mL^−1^ X-Gluc) at 37 °C in the dark for 12 h, and then decolorized with 70% ethanol for observation and photography.

For dual-luciferase assay, promoters were cloned into the pGreenII 0800-LUC reporter vector, and the CDS of *MaMYB12* or *MaMYB308* was cloned into the pGreenII 62-SK effector vector. The reporter and effector constructs were co-transformed into tobacco leaves. After 72 h of cultivation, 1 mmol·L^−1^ D-luciferin potassium salt was applied to the leaves, and luciferase activity was detected using a live imaging system. LUC and REN activities were measured using the Dual-Luciferase® Reporter Assay System (Promega, USA) following the manufacturer's protocol. REN activity, driven by the constitutive 35S promoter present on the same reporter vector, was used as an internal reference for normalizaion. Transcriptional activity is presented as the LUC/REN ratio. For each promoter, three independent transformation experiments were performed, each with three technical replicates.

### 2.8 Yeast one-hybrid (Y1H) assay

Promoter fragment of target gene was amplified from mulberry genomic DNA and cloned into the pAbAi vector (Clontech, USA), which was then linearized and integrated into the genome of the Y1HGold yeast strain to generate bait strains. The coding sequence of *MaMYB12* or *MaMYB308* was fused to the GAL4 activation domain in the pGADT7 vector (Clontech, USA) to generate prey plasmids. The resulting pGADT7-MYB construct was transformed into the corresponding bait yeast strains. Transformants were initially selected on SD/−Leu medium, and positive interactions were identified on SD/−Leu medium containing aureobasidin A (AbA) after incubation at 30 °C for 3–5 d. Empty pGADT7 vector was used as a negative control for each bait strain.

### 2.9 Electrophoretic mobility shift assay (EMSA)

EMSA was performed as previously described with minor modifications (Kang et al., 2024). Briefly, 5′-biotin-labeled DNA probes containing the MYB-binding motifs and their corresponding mutant probes were synthesized and annealed to form double-stranded DNA. Recombinant GST-MaMYB12 or GST-MaMYB308 proteins were incubated with biotin-labeled probe, along with unlabeled competitor probes or mutant probes, using a LightShift Chemiluminescent EMSA Kit (Coolaber Co. Ltd., Beijing, China) for 30 min at room temperature. The reaction mixtures were separated by 6% PAGE using 0.5 × Tris-borate-EDTA running buffer, and the shifted bands were detected by chemiluminescence.

### 2.10 Yeast two-hybrid (Y2H) assay

Full-length coding sequences of MaMYB308 and *MaMYB12* were cloned into pGBKT7 (BD, DNA-binding domain) and pGADT7 (AD, activation domain) vectors (Clontech, USA), respectively. Various combinations were co-transformed into the Y2HGold yeast strain. Transformants were selected on DDO (SD/−Leu/−Trp) medium. Protein-protein interactions were assessed by observing growth on QDO/X (SD/−Ade/-His/−Leu/−Trp with X-α-Gal) medium. The pairs AD + BD-53 and AD + BD-Lam were used as positive and negative controls, respectively.

### 2.11 Generation of transgenic mulberry hairy roots

The binary plasmid vector pLGNL, carrying the visual RUBY reporter gene (a gene cluster consisting of *CYP76AD6*, *CYP76AD1*, *BvDODA1*, and *cDOPA5GT*) was used for genetic transformation. The CDS of *MaMYB12* or *MaMYB308* was cloned into pLGNL (carrying a RUBY reporter gene), and the recombinant construct was transformed into *A. rhizogenes* strain K599. Hypocotyls of two-week-old tissue-cultured ‘Guisang You 62’ seedlings were wounded and inoculated with the bacterial suspension. Control seedlings were inoculated with *A. rhizogenes* K599 harboring the empty pLGNL vector to generate control hairy roots expressing solely the RUBY reporter gene. The inoculated seedlings were cultivated under a 16-h light/8-h dark photoperiod at 25 °C with a relative humidity of 50–60%. After approximately four weeks of cultivation, well-developed transgenic hairy roots were identified by the red betacyanin pigmentation produced by the RUBY reporter. For each construct, at least 10 independent transgenic root lines were established.

### 2.12 Antioxidant activity assays

For antioxidant activity assays, the same ethanol extracts prepared for flavonoid quantification as described above were used. DPPH radical scavenging activity was measured according to the method of Zhang et al. (2015). In brief, 0.1 mL of extract was mixed with 3.9 mL of DPPH solution (0.1 m mol·L^−1^ in methanol). The mixture was incubated in the dark for 30 min, and the absorbance was measured at 517 nm. The DPPH scavenging activity was calculated as: [(A_control- A_sample) / A_control] × 100%.

### 2.13 Data analysis

All experiments included at least three independent biological replicates. Experimental data are expressed as mean ± standard deviation (SD) from three independent biological replicates unless otherwise specified. Statistical differences were assessed by one-way ANOVA followed by Duncan's multiple range test using SPSS software. Differences were considered significant at *p* < 0.05. In all figures, asterisks (*) indicate significant differences compared to the control group, while different lowercase letters indicate significant differences among multiple groups (one-way ANOVA, *p* < 0.05).

## 3 Results

### 3.1 Identification and functional characterization of MYB transcription factors associated with flavonoid biosynthesis in mulberry

**Fig. 1:**
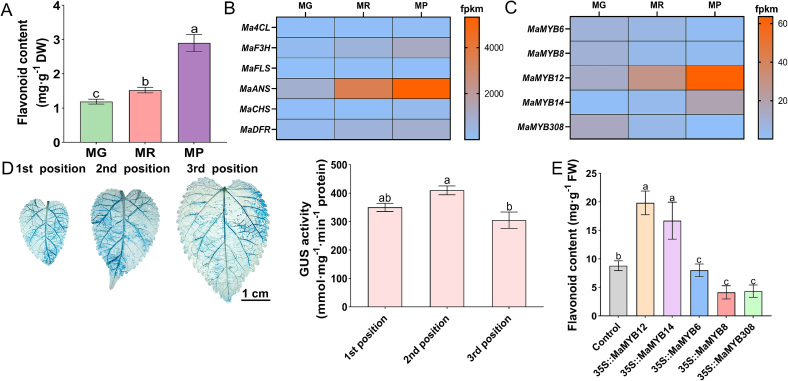
Screening and functional characterization of MYB transcription factors associated with flavonoid biosynthesis in mulberry. (A) Total flavonoid content in mulberry fruits at three developmental stages: MG (mature green, 20 days after flowering), MR (mature red, 30 days after flowering), and MP (mature purple, 40 days after flowering). Data are presented as mean ± SD (n = 3 biological replicates). Different lowercase letters indicate statistically significant differences between groups (*p* < 0.05). (B) Expression profiles of the key flavonoid biosynthetic genes (*Ma4CL*, *MaF3H*, *MaFLS*, *MaANS*, *MaCHS*, and *MaDFR*) in mulberry fruits at the MG, MR, and MP stages. (C) Expression profiles of five candidate MYB genes (*MaMYB6*, *MaMYB8*, *MaMYB12*, *MaMYB14*, and *MaMYB308*) in mulberry fruits at the MG, MR, and MP stages. (D) Optimization of the transient expression system in mulberry leaves. Left: GUS histochemical staining of leaves from different positions (1st, 2nd, 3rd from the apex) after infiltration with a 35S::GUS construct. Right: Quantitative GUS enzyme activity in the corresponding leaf. (E) Effect of transient overexpression of individual MYB genes on total flavonoid content in mulberry leaves. The empty vector was used as a control. Data are mean ± SD (*n* = 3 biological replicates). Different letters indicate statistically significant differences (*p* < 0.05). (For interpretation of the references to colour in this figure legend, the reader is referred to the web version of this article.)

Analysis of flavonoid content in mulberry fruits at different developmental stages showed a significant increase during ripening from the MG to MR and MP stages (Fig. 1A). Transcriptome data showed that the key flavonoid biosynthetic genes (*Ma4CL*, *MaF3H*, *MaFLS*, *MaANS*, *MaCHS*, and *MaDFR*) exhibited increased expression during fruit ripening (Fig. 1B). This expression pattern correlated positively with the accumulation of total flavonoids, suggesting that the flavonoid biosynthetic pathway is coordinately transcriptionally activated during mulberry fruit maturation.

Mining of transcriptome data identified 5 MYB genes (*MaMYB6*, *MaMYB8*, *MaMYB12*, *MaMYB14*, and *MaMYB308*) with expression profiles closely mirroring or opposing the flavonoid accumulation trend. *MaMYB12* and *MaMYB14* were upregulated during fruit maturation, whereas *MaMYB6*, *MaMYB8*, and *MaMYB308* were downregulated (Fig. 1C).

Phylogenetic analysis placed MaMYB12 within subgroup 7 (SG7) alongside known flavonol regulators such as *Arabidopsis* AtMYB12, while MaMYB14 was grouped with SG6 members. MaMYB6, MaMYB8, and MaMYB308 were clustered into SG4, which contains characterized transcriptional repressors (Supplementary Fig. S1A). Conserved motif analysis further revealed that MaMYB12 contains the SG7-specific conserved [W/x][L/x]LS motif (Czemmel et al., 2009), and MaMYB308 harbors the canonical C-terminal EAR repression motif (pdLNL[D/E]L) characteristic of SG4 repressors (Ohta et al., 2001) Supplementary Fig. S1B).

Given the absence of a stable genetic transformation system for mulberry, we employed a transient leaf-based expression system to assess the functions of the candidate MYBs. To optimize this transient expression system, mulberry leaves at different positions were infiltrated with *Agrobacterium* carrying a 35S::GUS construct. GUS histochemical staining and quantitative activity assays showed that GUS was robustly expressed in leaves at the first to third positions, with the second position leaves exhibiting the highest GUS activity (Fig. 1D). Therefore, leaves from the second position were used for all subsequent transient assays.

Transient overexpression of each candidate MYB in mulberry leaves revealed that *MaMYB12* and *MaMYB14* significantly increased total flavonoid content, with *MaMYB12* showing the strongest activating effect. In contrast, overexpression of *MaMYB6*, *MaMYB8*, and *MaMYB308* led to a significant reduction in total flavonoid content, with MaMYB308 acting as the most potent repressor (Fig. 1E). Based on these distinct and opposing phenotypic effects, MaMYB12 (activator) and MaMYB308 (repressor) were selected for detailed functional characterization.

### 3.2 MaMYB12 promotes flavonoid accumulation by activating key biosynthetic genes

To delineate the flavonoid metabolite profile regulated by *MaMYB12*, we performed targeted metabolomics on mulberry leaves transiently overexpressing *MaMYB12*. Compared with the empty-vector control, 30 flavonoid metabolites accumulated at significantly higher levels in *MaMYB12-*overexpressing leaves (Table 1). Glabranin (a dihydroflavone) showed the largest change, and six other compounds increased more than 10,000-fold. These included quercetin-7-O-(2″-malonyl)glucoside-5-O-glucoside (flavonol), kaempferol-7-O-glucoside (flavonol), kaempferol-3-O-(2”-*O*-acetyl)glucuronide (flavonol), luteolin-3’-O-glucoside (flavone), and isoliquiritin (chalcone). These results indicate that MaMYB12 exerts a broad promotive effect on flavonoid biosynthesis in mulberry.

**Table 1:** Significantly upregulated flavonoid metabolites in mulberry leaves following transient overexpression of *MaMYB12*. Metabolites were identified by UPLC-MS/MS coupled with a self-built flavonoid database (Lian Chuan Biotechnology Co., Ltd.), based on retention time and characteristic ion pair matching. Categories are based on structural characteristics of flavonoids (flavonols, dihydroflavones, flavones, chalcones, etc.). Leaves inoculated with *A. tumefaciens* GV3101 harboring the empty vector were used as controls. The peak area values are presented for control (Control) and *MaMYB12*-overexpressing (MaMYB12-OE) samples to show the original quantitative data underlying the fold change calculations. Fold change (MaMYB12-OE/Control) ≥ 2.0 and *p* < 0.05 was the criterion for significant upregulation. All values represent the mean of three independent biological replicates.

Compounds	Class II	Peak area		Log_2_ (MaMYB12-OE/Control)
		Control	MaMYB12-OE	
Isoliquiritin	Chalcone	9	96,665	13.39
Morachalcone B	Chalcone	163,230	644,400	1.98
Dihydrocharcone-4’-O-glucoside	Chalcone	84,596	180,300	1.09
Glabranine	Dihydroflavone	9	2,734,900	18.21
Licorice glycoside D2	Dihydroflavone	96,047	376,430	1.97
Bavachin	Dihydroflavone	850,340	2,837,600	1.74
Isobavachin	Dihydroflavone	850,340	2,837,600	1.74
Naringenin-7-O-glucoside (Prunin)	Dihydroflavone	387,150	927,090	1.26
Taxifolin-7-O-rhamnoside	Dihydroflavone	9	35,055	11.93
2,6,7,4’-Tetrahydroxyisoflavanone	Dihydroflavone	9	21,162	11.20
Delphinidin-3-O-(2”-O-glucosyl)rutinoside	Anthocyanin	9	42,719	12.21
Cyanidin-3-O-(2”-O-xylosyl)galactoside	Anthocyanin	9	39,048	12.08
Delphinidin-3,5,3′-Tri-O-glucoside	Anthocyanin	9	15,435	10.74
Malvidin-3,5-di-O-glucoside	Anthocyanin	9	7555	9.71
Luteolin-3’-O-glucoside	Flavonoid	9	218,100	14.56
Tectochrysin	Flavonoid	9	4073	8.82
Pinocembrin-7-O-(6”-O-malonyl)glucoside	Flavonoid	51,548	115,880	1.17
Quercetin-7-O-(2″-malonyl)glucosyl-5-O-glucoside	Flavonol	9	703,520	16.25
Kaempferol-7-O-glucoside	Flavonol	9	381,430	15.37
Kaempferol-3-O-(2”-*O*-acetyl)glucuronide	Flavonol	9	309,360	15.07
Kaempferol-3-O-glucorhamnoside	Flavonol	9	70,651	12.94
Quercetin-3-O-(2″‘-O-feruloyl)sophoroside	Flavonol	9	29,766	11.69
Kaempferol-3-O-(6″-p-Coumaroyl)galactoside	Flavonol	9	19,513	11.08
(2*R*,3*R*)-8-Prenyl-7,4′-dihydroxy-5-methoxydihydroflavonol	Flavonol	211,490	470,770	1.15
Apigenin-6-C-rhamnoside	Flavonoid Carbonoside	9	63,029	12.77
Catechin-5-O-glucoside	Flavanol	50,490	144,280	1.51
Sanggenol I	Other Flavonoid	9	9402	10.03
Kanzonol N	Other Flavonoid	9	4095	8.83
Sanggenol N	Other Flavonoid	5945	16,504	1.47
2’-Hydoxy,5-methoxyGenistein-4′,7-O-diglucoside	Isoflavone	20,149	45,970	1.19

**Fig. 2:**
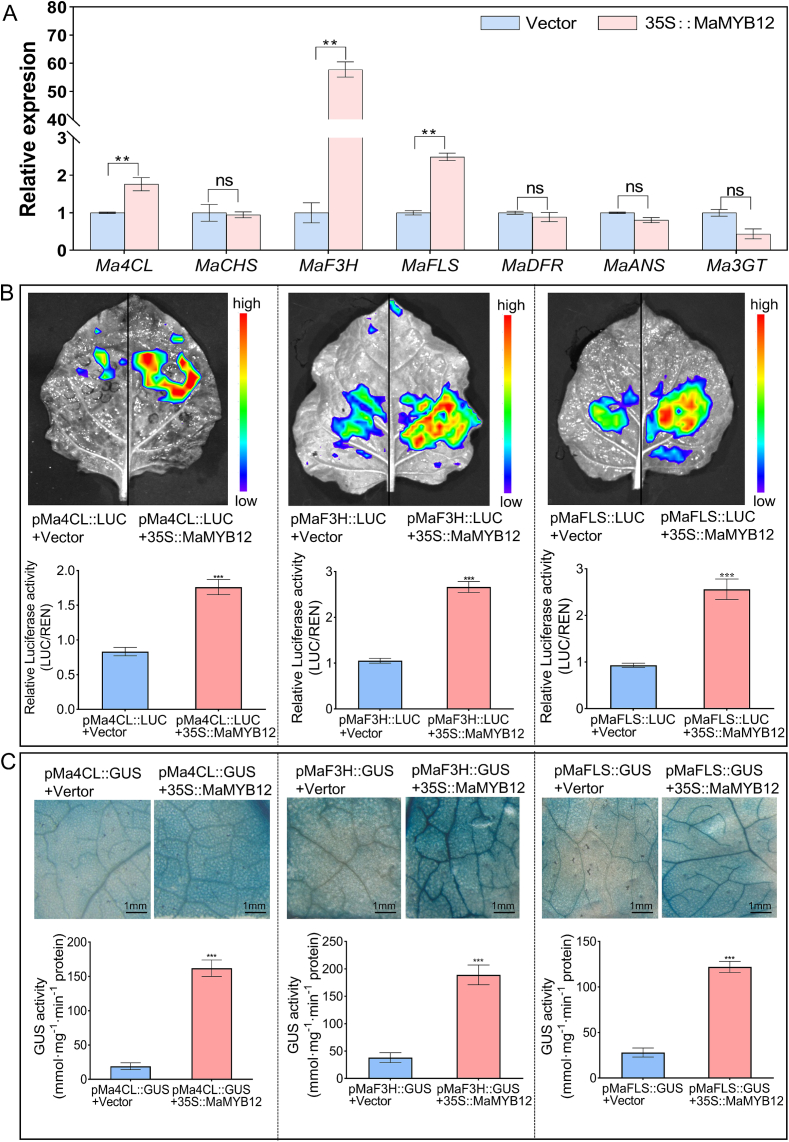
MaMYB12 activates key flavonoid biosynthetic genes. (A) Relative expression levels of flavonoid pathway genes after transient *MaMYB12* overexpression in mulberry leaves. The mulberry *ACTIN* gene was used for normalization. Data are mean ± SD (*n* = 3 biological replicates). Double asterisks indicate a significant difference compared to the empty vector control (*p* < 0.01). (B) Luciferase reporter assay confirming promoter activation by MaMYB12. Quantified luminescence intensity presented as the LUC/REN ratio. Data are mean ± SD (n = 3 biological replicates). Three asterisks indicate a significant difference (*p* < 0.001). (C) GUS histochemical staining assay showing MaMYB12-mediated activation of target gene promoters in *N. benthamiana* leaves. Data are mean ± SD (*n* = 3 biological replicates). Three asterisks indicate a significant difference (*p* < 0.001).

To understand the molecular mechanism underlying this promotional effect, the expression levels of key structural genes in the flavonoid pathway were examined. qRT-PCR analysis showed that overexpression of *MaMYB12* specifically upregulated the transcript levels of the upstream gene *Ma4CL*, *MaF3H*, and the key flavonol branch gene *MaFLS*, while the expression of *MaCHS*, *MaDFR*, anthocyanidin synthase gene (*MaANS*), and anthocyanidin 3-O-glucosyltransferase (Ma3GT) remained unchanged (Fig. 2A). Luciferase reporter and GUS staining assays confirmed that MaMYB12 protein directly activates the promoters of *Ma4CL*, *MaF3H*, and *MaFLS* (Fig. 2B, C).

**Fig. 3:**
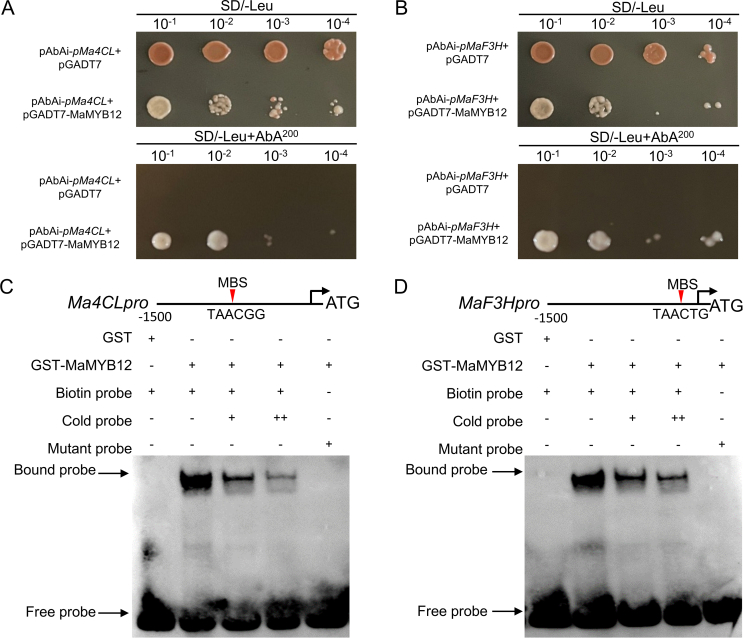
Y1H assays and EMSA confirm the direct binding of MaMYB12 to the target gene promoters. (A, B) Yeast one-hybrid (Y1H) assays validating the direct interaction between MaMYB12 and the target gene promoters of *Ma4CL* (*pMa4CL*, A) and *MaF3H* (*pMaF3H*, B). Yeast cells co-transformed with the pGADT7-MaMYB12 prey vector and the pAbAi bait vector containing the respective promoters were grown on selective medium lacking leucine and containing aureobasidin A (AbA). Growth indicates a positive interaction. The empty pGADT7 vector was used as a negative control. (C, D) Electrophoretic mobility shift assay (EMSA) demonstrating the direct binding of recombinant GST-MaMYB12 protein to biotin-labeled probes derived from the *Ma4CL* (C) and *MaF3H* (D) promoters. The shifted bands, representing protein-DNA complexes, are indicated by arrows. Competition assays were performed with excess unlabeled specific probe (cold probe) to confirm binding specificity.

Prediction results using PlantCARE software showed that the promoter sequences of *Ma4CL* and *MaF3H* contain MYB transcription factor binding sites. To further validate the direct interaction between MaMYB12 and the promoters of *Ma4CL* and *MaF3H*, Yeast one-hybrid (Y1H) assays were performed and validated the direct binding of MaMYB12 to the promoters of *Ma4CL* and *MaF3H* (Fig. 3A, B). EMSA were also performed using recombinant GST-MaMYB12 protein and biotin-labeled probes containing MYB-binding motifs from the target promoters. The results showed that MaMYB12 protein specifically bound to the probes derived from *Ma4CL* and *MaF3H* promoters, respectively, resulting in the formation of shifted bands (Fig. 3C, D). These results collectively establish MaMYB12 as a direct upstream activator of *Ma4CL* and *MaF3H*.

### 3.3 MaMYB308 broadly suppresses flavonoid biosynthesis

In contrast to *MaMYB12*, *MaMYB308* functioned as a repressor of flavonoid accumulation in mulberry. Targeted metabolomics analysis showed that overexpression of *MaMYB308* led to a significant reduction in the content of 106 flavonoid metabolites compared with the empty vector control (Table 2). Luteolin-7-O-glucoside showed the most significant reduction, and 7 other compounds decreased by more than 10,000-fold, including eriodictyol-3’-O-glucoside (dihydroflavone), epicatechin glycoside (flavanol), quercetin-3-O-xyloside (flavonol), quercetin-3-O-(6”-O-p-coumaroyl) glucoside (flavonol), quercetin-3-O-(2”-O-p-coumaroyl) galactoside (flavonol), and avicularin (flavonol). The suppressed metabolites covered almost all flavonoid subclasses, including flavonols, flavones, flavanones, dihydroflavonols, and anthocyanins, indicating that *MaMYB308* exerts a broad inhibitory effect on the entire flavonoid metabolic network in mulberry.

**Table 2:** Significantly downregulated flavonoid metabolites in mulberry leaves following transient overexpression of *MaMYB308*. Metabolite identification and classification methods are as described in Table 1. Leaves inoculated with *A. tumefaciens* GV3101 harboring the empty vector were used as controls. The peak area values are presented for control (Control) and *MaMYB308*-overexpressing (MaMYB308-OE) samples to show the original quantitative data underlying the fold change calculations. Downregulated metabolites were defined as those with fold change (MaMYB308-OE/Control) ≤ 0.5 and *p* < 0.05. All values represent the mean of three independent biological replicates.

Compounds	Class II	Peak area		Log_2_ (MaMYB308-OE/Control)
		Control	MaMYB308-OE	
4′-hydroxy-2,4,6-trimethoxydihydrochalcone	Chalcone	60,608	9	−12.72
Naringin Dihydrochalcone	Chalcone	801,100	79,727	−3.33
Isobavachalcone D	Chalcone	875,870	135,720	−2.69
Okanin	Chalcone	15,703,000	4,926,000	−1.67
Eriodictyol-3’-O-glucoside	Flavanone	266,490	9	−14.85
C-glucosyl-C-arabinosyl-2-hydroxynaringenin	Flavanone	7874	9	−9.77
2-hydroxynaringenin	Flavanone	6,489,900	710,290	−3.19
Hesperetin-7-O-rutinoside (Hesperidin)	Flavanone	61,842	16,546	−1.90
Leachianone G	Flavanone	190,810	62,240	−1.62
Hesperetin-7-O-glucoside	Flavanone	19,029,000	6,465,600	−1.56
Eriodictyol (5,7,3′,4’-Tetrahydroxyflavanone)	Flavanone	21,801,000	7,660,100	−1.51
Hesperetin-5-O-glucoside	Flavanone	33,046,000	15,931,000	−1.05
Silibinin	Flavanonol	41,714	17,160	−1.28
Dihydromyricetin (Ampelopsin)	Flavanonol	95,691	46,305	−1.05
Dihydrokaempferol-7-O-glucoside	Flavanonol	908,090	447,280	−1.02
5,7-Dihydroxy-2′-methoxy-3′,4′-methyleneoxydihydroisoflavone	Flavanonol	28,749	12,494	−1.20
Delphinidin-3-O-arabinoside	Anthocyanidin	70,675	9	−12.94
Delphinidin-3-O-(6”-O-p-coumaroyl)glucoside	Anthocyanidin	7,245,200	2,037,400	−1.83
Cyanidin-3-O-(2”-O-glucosyl)rutinoside	Anthocyanidin	130,120	49,725	−1.39
Luteolin-7-O-glucoside (Cynaroside)	Flavone	1,135,000	9	−16.94
Chrysin-7-O-glucoside	Flavone	25,725	9	−11.48
Genkwanin (Apigenin 7-methyl ether)	Flavone	8361	9	−9.86
Apigenin-7-O-glucuronide	Flavone	314,040	24,490	−3.68
Apiin	Flavone	76,040	14,884	−2.35
Isoscutellarein	Flavone	2,044,700	438,280	−2.22
Luteolin-7-O-glucuronide	Flavone	606,360	131,900	−2.20
Luteolin (5,7,3′,4’-Tetrahydroxyflavone)	Flavone	2,130,300	468,620	−2.18
Nepetin (5,7,3′,4’-Tetrahydroxy-6-methoxyflavone)	Flavone	400,380	110,900	−1.85
Tamarixetin-3-O-(6″-malonyl)glucoside	Flavone	341,030	100,940	−1.76
7,4’-Dihydroxyflavone	Flavone	8079	2426	−1.74
Chrysoeriol-7-O-rutinoside	Flavone	154,190	51,540	−1.58
6-Hydroxyluteolin 5-glucoside	Flavone	109,280,000	38,505,000	−1.50
Hispidulin (5,7,4′-Trihydroxy-6-methoxyflavone)	Flavone	20,758	7577	−1.45
Rhamnocitrin (7-Methylkaempferol)	Flavone	25,726	9465	−1.44
Luteolin-7-O-(6″-malonyl)glucoside-5-O-rhamnoside	Flavone	209,820	77,777	−1.43
Tetahydroxyflavone-7-O-glucuronide	Flavone	31,887	13,752	−1.21
5,2’-Dihydroxy-7,8-dimethoxyflavone glycosides	Flavone	482,360	218,670	−1.14
Chrysoeriol-7-O-(6″-acetyl) glucoside	Flavone	1,168,700	534,190	−1.13
5,7,2′-Trhiyroxy-8-methoxyflavone	Flavone	57,727	27,370	−1.08
Luteolin-7-O-(6″-eudesmyl)glucoside	Flavone	127,590	62,689	−1.03
Tamarixetin-3-O-rutinoside	Flavone	19,165	9477	−1.02
Apigenin-4’-O-glucoside	Flavone	110,350	54,830	−1.01
Apigenin-7-O-glucoside(Cosmosiin)	Flavone	110,350	54,830	−1.01
Apigenin-7-O-α-D-glucopyranoside	Flavone	110,350	54,830	−1.01
6-Hydroxyluteolin	Flavone	377,020	187,500	−1.01
Quercetin-3-O-xyloside (Reynoutrin)	Flavonol	167,900	9	−14.19
Quercetin-3-O-(6”-O-p-Coumaroyl)glucoside	Flavonol	161,860	9	−14.13
Quercetin-3-O-(2”-O-p-Coumaroyl)galactoside	Flavonol	161,860	9	−14.13
Avicularin	Flavonol	134,780	9	−13.87
Kaempferol-3-O-sambubioside	Flavonol	169,740	14,159	−3.58
Kaempferol-3-O-(6″-Malonyl)glucoside-7-O-Glucoside	Flavonol	1,728,000	324,770	−2.41
Myricetin	Flavonol	473,110	93,770	−2.33
Kaempferol-3-O-(6″-acetyl)glucoside	Flavonol	1,581,100	328,310	−2.27
Kaempferol-3-O-(6″-malonyl)galactoside	Flavonol	3,252,500	678,340	−2.26
Kaempferol-3-O-glucuronide	Flavonol	528,890	122,980	−2.10
Quercetin-3-O-sambubioside	Flavonol	184,960	43,332	−2.09
Quercetin-3-O-(4”-O-glucosyl)rhamnoside	Flavonol	8,651,900	2,121,500	−2.03
Isorhamnetin	Flavonol	37,841	10,001	−1.92
Quercetin-4’-O-glucoside (Spiraeoside)	Flavonol	51,462,000	14,256,000	−1.85
Quercetin-3-O-(6”-*O*-acetyl)galactoside	Flavonol	9,398,400	2,731,700	−1.78
Quercetin-3-O-rutinoside (Rutin)	Flavonol	12,183,000	3,551,000	−1.78
Kaempferol-3-O-(6″-malonyl)glucoside	Flavonol	1,117,000	325,740	−1.78
Isorhamnetin-3-O-(6″-malonyl)glucoside	Flavonol	265,580	78,531	−1.76
Quercetin-7-O-(6″-malonyl)glucoside	Flavonol	21,833,000	6,748,800	−1.69
Quercetin-3-O-rhamnoside(Quercitrin)	Flavonol	121,520	38,755	−1.65
Kaempferol-3-O-neohesperidoside-7-O-glucoside	Flavonol	127,310	42,381	−1.59
Rhamnetin	Flavonol	126,860	42,692	−1.57
Isorhamnetin-3-O-neohesperidoside	Flavonol	64,202	22,050	−1.54
Quercetagetin; 3,3′,4′,5,6,7-Hexahydroxyflavone	Flavonol	501,140	176,300	−1.51
3-O-Methylquercetin	Flavonol	98,816	35,112	−1.49
Kaempferol-3-O-(2″-acetyl)glucoside	Flavonol	94,880	34,068	−1.48
Kaempferol-3-O-sophoroside-7-O-rhamnoside	Flavonol	130,120	49,725	−1.39
6-Hydroxykaempferol-7-O-glucoside	Flavonol	100,950,000	41,565,000	−1.28
Quercetin-3-O-(6”-*O*-acetyl)glucoside	Flavonol	785,000	323,350	−1.28
Quercetin	Flavonol	1,237,000	528,920	−1.23
Isorhamnetin-7-O-glucoside (Brassicin)	Flavonol	908,190	391,680	−1.21
Quercetin-7-O-rutinoside-4’-O-glucoside	Flavonol	99,832	43,873	−1.19
Quercetin-3-O-rutinoside-7-O-glucoside	Flavonol	99,832	43,873	−1.19
Morin	Flavonol	2,073,500	947,750	−1.13
Quercetin-5-O-β-D-glucoside	Flavonol	82,826,000	40,170,000	−1.04
Sexangularetin-3-O-glucoside-7-O-rhamnoside	Flavonol	54,326	26,732	−1.02
Chrysoeriol-6-C-glucoside (Isoscoparin)	Flavonoid arbonoside	68,099	9	−12.89
Luteolin-8-C-arabinoside	Flavonoid carbonoside	41,746	9	−12.18
Apigenin-6,8-di-C-glucoside (Vicenin-2)	Flavonoid carbonoside	27,762	9049	−1.62
Apigenin-6-C-(2″-glucosyl)arabinoside	Flavonoid carbonoside	24,055	8850	−1.44
Epicatechin glucoside	Flavanol	191,760	9	−14.38
Catechin	Flavanol	516,800	71,707	−2.85
Epicatechin	Flavanol	1,587,800	220,890	−2.85
Epicatechin-3’-O-β-D-glucopyranoside	Flavanol	619,430	106,650	−2.54
Epicatechin-6-C-β-D-glucopyranoside	Flavanol	613,800	107,630	−2.51
Tetrahydroxyflavan-(4α-8-epicatechin)	Flavanol	61,747	14,185	−2.12
Cinchonain Id	Flavanol	531,470	138,460	−1.94
5,7,3′,4′,5’-Pentahydroxyflavan (Tricetiflavan)	Flavanol	52,768	16,338	−1.69
Epicatechin-4’-O-β-D-glucopyranoside	Flavanol	5,299,900	1,852,600	−1.52
Sanggenon L	Other Flavonoid	11,300	9	−10.29
Kuwanon P	Other Flavonoid	104,400	30,023	−1.80
Moracin D	Other Flavonoid	256,740	98,039	−1.39
3,3’-*O*-Dimethylellagic Acid	Tannin	7889	9	−9.78
Ginnalin A (2,6-Di-O-Galloyl-1,5-Anhydro-*D*-Glucitol)	Tannin	94,296	20,331	−2.21
2-Hydroxy-2,3-dihydrogenistein	Isoflavone	6,900,000	694,080	−3.31
6”-O-Malonylgenistin	Isoflavone	171,650	17,831	−3.27
2’-Hydroxygenistein	Isoflavone	2,001,700	440,660	−2.18
Wighteone	Isoflavone	248,950	56,984	−2.13
2’-Hydoxy,5-methoxyGenistein-O-rhamnosyl-glucoside	Isoflavone	58,443	28,042	−1.06
2α,3α-Epoxy-5,7,3′,4′-tetrahydroxyflavan-(4β-8-catechin)	Proanthocyanidin	57,856	9	−12.65
2α,3α-Epoxy-5,7,3′,4′-tetrahydroxyflavan-(4β-8-epicatechin)	Proanthocyanidin	94,125	13,042	−2.85

**Fig. 4:**
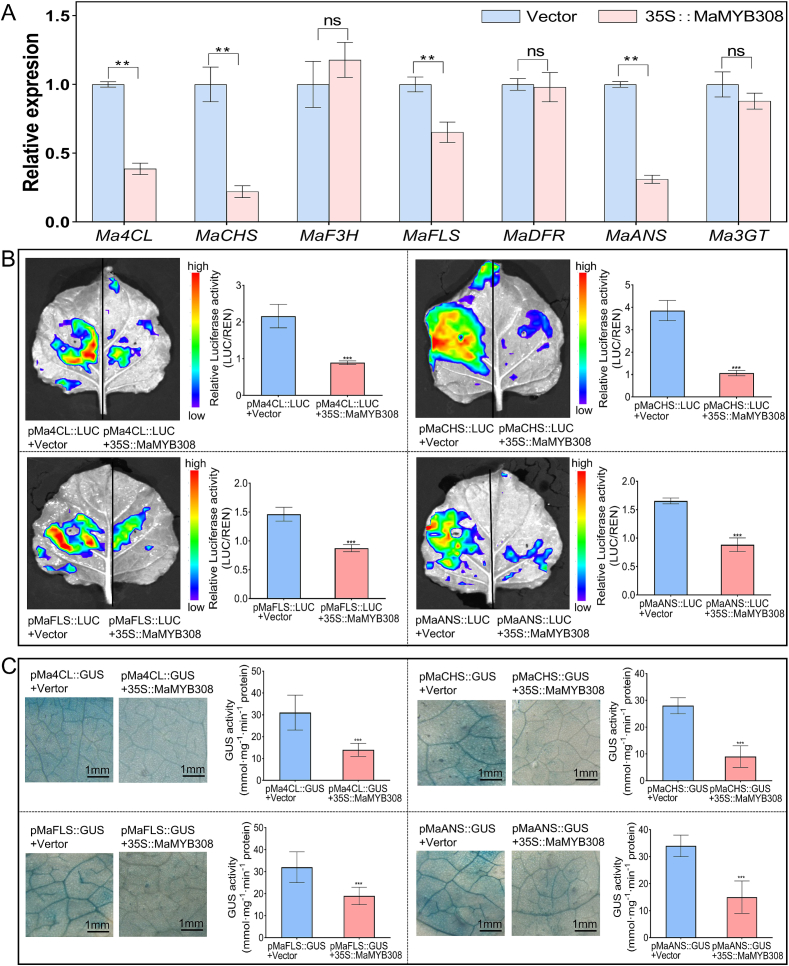
MaMYB308 represses key flavonoid biosynthetic genes. (A) Relative expression levels of flavonoid pathway genes after transient *MaMYB308* overexpression in mulberry leaves. The mulberry *ACTIN* gene was used for normalization. Data are mean ± SD (*n* = 3 biological replicates). Double asterisks indicate a significant difference compared to the empty vector control (*p* < 0.01). (B) Luciferase reporter assay confirming promoter repression by MaMYB308. Quantified luminescence intensity presented as the LUC/REN ratio. Data are mean ± SD (n = 3 biological replicates). Three asterisks indicate a significant difference (*p* < 0.001). (C) GUS histochemical staining assay showing MaMYB308-mediated repression of target gene promoters in *N. benthamiana* leaves. Data are mean ± SD (n = 3 biological replicates). Three asterisks indicate a significant difference (*p* < 0.001).

Consistent with the metabolomic data, qRT-PCR analysis showed that transient overexpression of *MaMYB308* significantly downregulated the expression levels of *Ma4CL*, *MaCHS*, *MaFLS*, and *MaANS*, while the expression of other structural genes in the flavonoid pathway were unaffected (Fig. 4A). This result suggests that *MaMYB308* inhibits both upstream and downstream structural genes in the flavonoid pathway. Luciferase reporter assays and GUS histochemical staining further confirmed that MaMYB308 significantly suppressed the transcriptional activity of the promoters of *Ma4CL*, *MaCHS*, *MaFLS*, and *MaANS* (Fig. 4B, C).

**Fig. 5:**
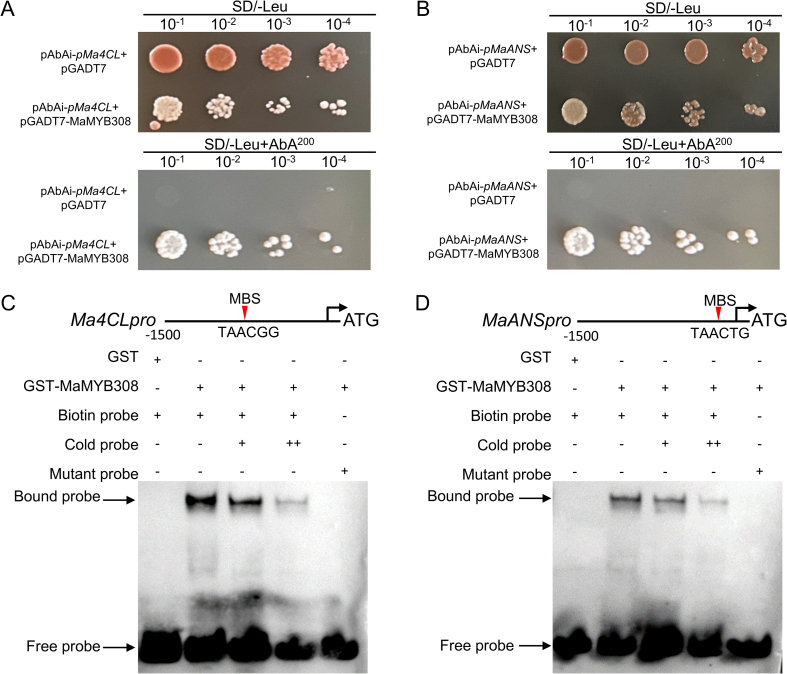
Y1H and EMSA assays confirm the direct binding of MaMYB308 to target gene promoters. (A, B) Y1H assays validating the direct interaction between MaMYB308 and the promoters of *Ma4CL* (*pMa4CL*, A) and *MaANS* (*pMaANS*, B). (C, D) EMSA demonstrating the direct binding of recombinant GST-MaMYB308 protein to biotin-labeled probes from the *Ma4CL* (C) and *MaANS* (D) promoters. The shifted bands are indicated by arrows. Competition assays with excess unlabeled specific probe (cold probe) confirm binding specificity.

Prediction results using PlantCARE software showed that the promoter sequences of *Ma4CL* and *MaANS* contain MYB transcription factor binding sites. To further validate the direct interaction between Yeast one-hybrid (Y1H) assays further confirmed the direct binding of MaMYB308 to the promoters of *Ma4CL* and *MaANS* (Fig. 5A, B). The direct binding of MaMYB308 to the promoters was further verified via EMSA assay. The results also confirmed these interactions, demonstrating that recombinant GST-MaMYB308 protein specifically bound to probes derived from the *Ma4CL* and *MaANS* promoters, respectively (Fig. 5C, D). These results establish MaMYB308 as a direct transcriptional repressor of *Ma4CL* and *MaANS*.

### 3.4 MaMYB12 and MaMYB308 exhibit transcriptional antagonism without direct protein-protein interaction

**Fig. 6:**
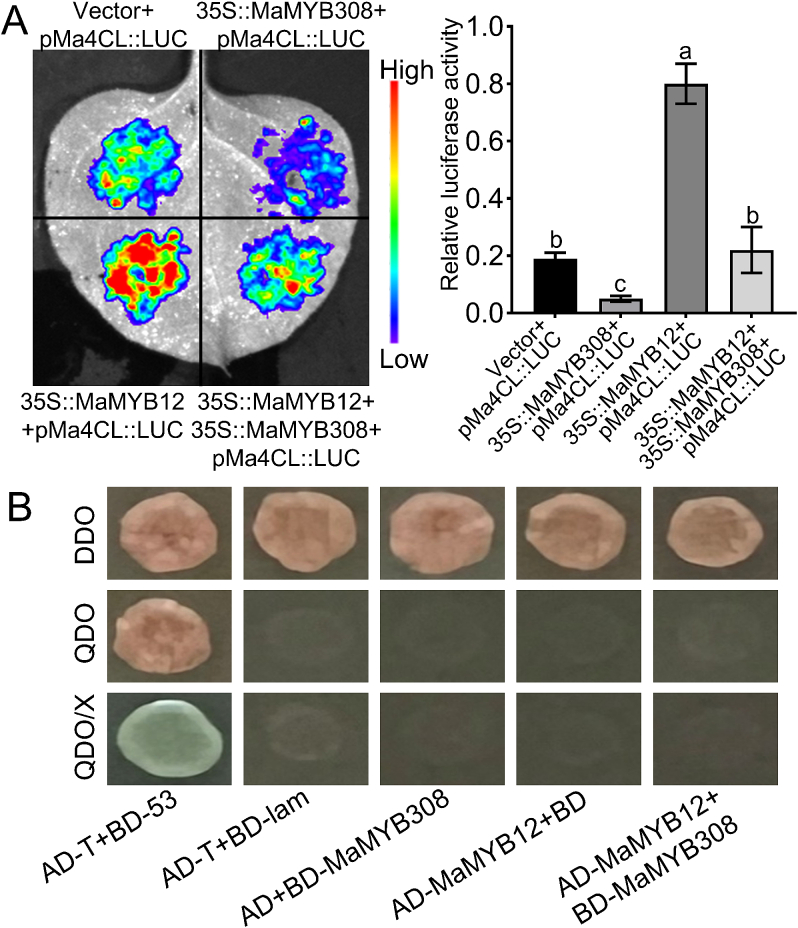
MaMYB12 and MaMYB308 exhibit transcriptional antagonism through competitive binding *Ma4CL* promoter. (A) Dual-luciferase assays showing the antagonistic effect of MaMYB308 on MaMYB12-mediated activation and MaMYB12 on MaMYB308-mediated repression of the *Ma4CL* promoter. Quantified luminescence intensity presented as the LUC/REN ratio. Data are mean ± SD (n = 3 biological replicates). Different letters indicate statistically significant differences (*p* < 0.05). (B) Yeast two-hybrid (Y2H) assay to test for direct protein-protein interaction between MaMYB12 and MaMYB308.

EMSA analysis showed that both MaMYB308 and MaMYB12 could directly bind to the promoter of the *Ma4CL* gene and shared the same binding site. To directly verify the transcriptional antagonism between MaMYB12 and MaMYB308, dual-luciferase co-transfection assays were performed in tobacco leaves. MaMYB12 and MaMYB308 effector vectors were mixed at a 1:1 ratio and co-infiltrated with reporter vectors carrying the *Ma4CL* promoter. Compared with the single *MaMYB12-*overexpressing group, the co-expression of *MaMYB12 and MaMYB308* significantly reversed the MaMYB12-mediated activation of the target promoter. By contrast, compared with the group overexpressing *MaMYB308* alone, co-expression of the two genes significantly reversed the MaMYB308-mediated repression of target promoter (Fig. 6A). These results confirmed the transcriptional antagonism between MaMYB308 and MaMYB12.

To explore whether the antagonism is mediated by protein-protein interaction, yeast two-hybrid (Y2H) assays were performed. No positive colonies were observed for the pairwise combinations of MaMYB12 and MaMYB308 (AD-MaMYB12 + BD-MaMYB308) on QDO medium with X-α-Gal, while the positive control (AD-T + BD-53) grew well and turned blue (Fig. 6B). These results indicate that MaMYB12 and MaMYB308 do not interact directly at the protein level, and their antagonistic effect is likely achieved through competition for binding sites on target gene promoters.

### 3.5 Antagonistic roles of MaMYB12 and MaMYB308 in stable transgenic mulberry hairy roots

**Fig. 7:**
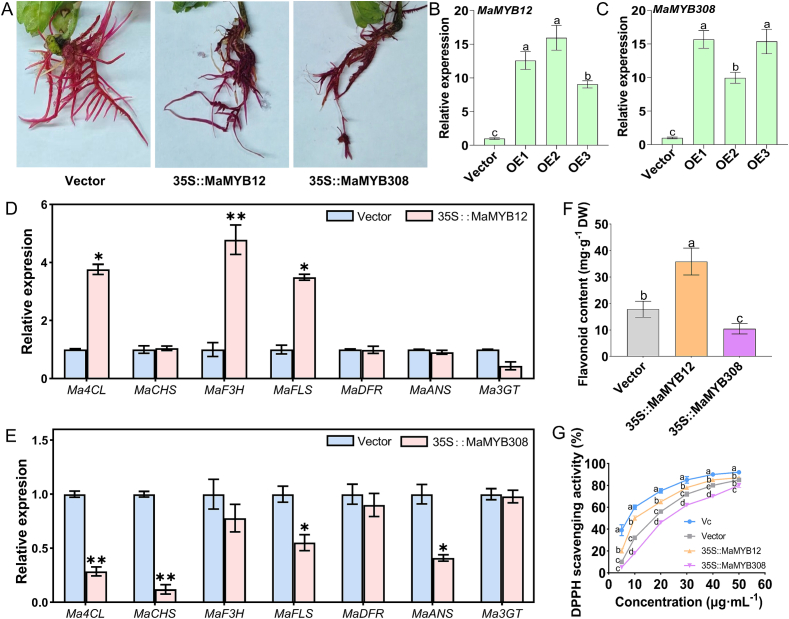
Functional validation of MaMYB12 and MaMYB308 in stable transgenic mulberry hairy roots. (A) Representative images of transgenic hairy roots expressing the RUBY reporter gene, showing red betacyanin pigmentation. (B, C) Relative expression levels of *MaMYB12* (B) and *MaMYB308* (C) in independent transgenic hairy root lines (OE1, OE2, OE3) compared to the vector (the empty pLGNL vector carrying RUBY reporter gene), as determined by qRT-PCR. The mulberry *ACTIN* gene was used for normalization. (D, E) Relative expression levels of target flavonoid biosynthetic genes in *MaMYB12*-overexpressing (35S::MaMYB12) lines (D) and *MaMYB308*-overexpressing (35S::MaMYB12) lines (E). (F) Total flavonoid content in the transgenic hairy root lines. Data are mean ± SD (n = 3 biological replicates). Different letters denote significant differences (*p* < 0.05). (G) DPPH radical scavenging activity of extracts from the transgenic hairy root lines. Data are mean ± SD (n = 3 biological replicates). Different letters indicate statistically significant differences (*p* < 0.05). (For interpretation of the references to colour in this figure legend, the reader is referred to the web version of this article.)

To definitively confirm the regulatory functions of MaMYB12 and MaMYB308, stable transgenic mulberry hairy roots overexpressing *MaMYB12* or *MaMYB308* were established. Transgenic hairy roots were first verified by the red betacyanin pigments in the roots produced by the RUBY reporter gene. Hairy roots expressing the RUBY reporter gene showed intense red pigmentation, indicating high transgene activity (Fig. 7A). qRT-PCR analysis further confirmed that transcript levels of *MaMYB12* and *MaMYB308* were significantly elevated in their respective transgenic lines compared to the control (Fig. 7B, C), demonstrating the successful establishment of stable overexpression lines.

Analysis of the expression of key flavonoid biosynthetic genes in these hairy roots showed that *MaMYB12*-overexpressing lines exhibited significantly upregulated transcript levels of *Ma4CL*, *MaF3H*, and *MaFLS*, while *MaMYB308*-overexpressing lines showed significantly downregulated expression of *Ma4CL*, *MaCHS*, *MaFLS*, and *MaANS* (Fig. 7D, E). These results further confirm the antagonistic roles of MaMYB12 and MaMYB308 in regulating flavonoid biosynthesis in a stable genetic context, and validate the utility of the hairy root system as a metabolic engineering platform for mulberry.

Consistent with the results of transient overexpression in mulberry leaves, hairy roots overexpressing *MaMYB12* accumulated significantly higher levels of flavonoids relative to the control. In contrast, overexpression of *MaMYB308* resulted in a marked decrease in flavonoid content (Fig. 7F). These results further confirm the antagonistic roles of MaMYB12 and MaMYB308 in regulating flavonoid biosynthesis in a stable genetic context, and validate the utility of the hairy root system as a metabolic engineering platform for mulberry.

To directly link the genetic modification of mulberry to its functional food properties, the antioxidant activity of extracts from the transgenic hairy roots were assessed using DPPH assays. It was showed that *MaMYB12*-overexpressing lines exhibited significantly enhanced DPPH radical scavenging activity compared with the control, while *MaMYB308*-overexpressing lines showed reduced activity (Fig. 7G). These results demonstrate that manipulation of the MaMYB12-MaMYB308 regulatory module directly affects the antioxidant potential of mulberry tissues, supporting its relevance for functional food development.

## 4 Discussion

This study identifies MaMYB12 (SG7) and MaMYB308 (SG4) as antagonistic regulators of flavonoid biosynthesis in mulberry, with implications for optimizing food ingredients from mulberry. These findings not only uncover a key transcriptional module governing flavonoid metabolism in a woody plant, but also provide actionable genetic targets for molecular breeding of mulberry varieties with optimized flavonoid content and composition for functional food applications.

### 4.1 Antagonistic regulatory functions and molecular mechanisms of MaMYB12 and MaMYB308

Phylogenetic and motif analyses clearly classified MaMYB12 and MaMYB308 into SG7 and SG4, respectively, which is consistent with their activator and repressor functions that have been reported in model plants (Jin et al., 2000; Li et al., 2016; Stracke et al., 2007). This distinct phylogenetic and structural divergence is the molecular basis for their opposing regulatory roles in the flavonoid metabolic network. In herbaceous model plants such as *Arabidopsis* and *Vitis vinifera*, SG7 MYBs (e.g., AtMYB12, VvMYBF1) are highly specific for the flavonol biosynthetic branch, only activating *FLS* and early pathway genes (Czemmel et al., 2009; Stracke et al., 2007), while SG4 repressors (e.g., AtMYB4, VvMYBC2-L2) mainly target early phenylpropanoid or flavonoid pathway genes to maintain the metabolic homeostasis of plant (Xie et al., 2019; Yoshida et al., 2015). For example, AtMYB4 represses *C4H* expression to control sinapate ester biosynthesis (Jin et al., 2000), while VvMYBC2-L1 in grapevine represses both early and late anthocyanin pathway genes (Xie et al., 2019). In poplar (*Populus trichocarpa*), MYB182 represses proanthocyanidin and anthocyanin biosynthesis by downregulating multiple structural genes (Yoshida et al., 2015). In contrast, our study reveals that MaMYB12 has a broader regulatory spectrum in mulberry, activating *Ma4CL*, *MaF3H* and *MaFLS* to promote the accumulation of flavonols, dihydroflavones, chalcones and anthocyanins, rather than being limited to the flavonol branch. MaMYB308 acts as a global repressor that directly targets both early (*Ma4CL*, *MaCHS*) and late (*MaFLS*, *MaANS*) flavonoid pathway genes. This unique regulatory mechanism is likely an adaptive evolution of mulberry, may represent an adaptive strategy to finely control flavonoid synthesis in response to complex developmental and environmental cues, differing from the more specialized regulation seen in some annuals.

Overexpression of *MaMYB12* significantly promoted the accumulation of 30 flavonoid metabolites in mulberry leaves, including flavonols, dihydroflavones, chalcones, and certain anthocyanins. This result indicates that MaMYB12 has a broader regulatory spectrum in mulberry compared to its *Arabidopsis* homolog *AtMYB12*, which is specific to flavonols (Wang et al., 2016), likely reflecting the complexity of the metabolic network in mulberry that produces a diverse array of compounds. By activating *Ma4CL*, an upstream gene, MaMYB12 can influence flux into multiple downstream branches. Conversely, MaMYB308, harboring a canonical EAR repression motif, functioned as a global suppressor that downregulates both early and late pathway genes and drastically reducing nearly all flavonoid subclasses. Notably, MaMYB308 repressed the accumulation of 106 flavonoid metabolites, while MaMYB12 only activated 30 metabolites, indicating that the repressor has a substantially broader regulatory scope. This difference may be explained by the fact that MaMYB308 targets multiple key nodes in the flavonoid pathway (including early genes *Ma4CL* and *MaCHS*, and late genes *MaFLS* and MaANS), effectively blocking the metabolic flux at both the entry and branching points of the flavonoid network. In contrast, MaMYB12 only targets three genes (*Ma4CL*, *MaF3H* and *MaFLS*), which may allow for more directed metabolic flux toward specific flavonoid subclasses. Additionally, the promoters of genes repressed by MaMYB308 contain multiple MYB-binding sites compared to those activated by MaMYB12, which may enable MaMYB308 to exert stronger and broader transcriptional repression effects. The ability of MaMYB308 to regulate *MaCHS*, a key gene that commits carbon flux to the entire flavonoid pathway, may be the main reason for its global suppressive effect on all downstream flavonoid subclasses. This potent repression likely plays a crucial role in preventing metabolic overburden in mulberry and redirecting carbon resources to other metabolic pathways during specific developmental stages or under certain environmental conditions.

The direct functional antagonism between MaMYB12 and MaMYB308 provides a molecular basis for the precise spatiotemporal regulation of flavonoid metabolism in mulberry. Their inverse expression patterns during fruit development—rising *MaMYB12* and declining *MaMYB308*—correlate with the observed increase in flavonoid content during ripening. This inverse expression pattern of the activator and repressor during fruit development likely contributes to the dynamic increase in flavonoid content observed during fruit ripening. Decreased expression of transcriptional repressors relieves their transcriptional repression of genes involved in the flavonoid biosynthetic pathway, whereas increased expression of transcriptional activators drives the transcriptional activation of these pathway genes. The coordinated action of this “activator-repressor” pair likely enables mulberry to flexibly respond to developmental signals and environmental changes, and dynamically adjust the flux of flavonoid synthesis. The identification of these two key regulatory factors deepens our understanding of secondary metabolic regulatory networks in woody plants, and provides key genetic targets for metabolic engineering strategies to directionally enhance the synthesis of high-value flavonoid products in mulberry.

### 4.2 Implications for functional food development and molecular breeding

Mulberry is an underutilized source of flavonoid-based functional food ingredients. Our findings enable targeted breeding strategies to enhance flavonoid yields. Overexpressing *MaMYB12* or knocking out *MaMYB308* could effectively increase the content of bioactive flavonoids in mulberry leaves, fruits or roots. This would improve the nutritional value of mulberry-derived products and potentially reduce production costs for flavonoid-based nutraceuticals. The transgenic hairy root system established in this study serves as a scalable “plant biofactory” for flavonoid production, which avoids the long generation time of woody plants and the limitations of field cultivation. This platform can be used to optimize flavonoid profiles for specific functional food applications and accelerate the breeding progress of high-flavonoid mulberry varieties.

Future research should focus on four core directions to translate these findings into functional food applications. First, systematically assessing the bioavailability in vivo and health-promoting effects of flavonoids from *MaMYB12*-overexpressing mulberry through preclinical animal models and human intervention trials. Second, developing high-throughput marker-assisted breeding tools to introgress *MaMYB12* overexpression or *MaMYB308* knockout traits into commercial mulberry varieties. Third, deciphering the interaction network between MaMYB12/MaMYB308 and other key regulators such as basic Helix-Loop-Helix (bHLH) and WD40 repeat domain (WD40) proteins. Fourth, investigating whether MaMYB308 affects other branches of the phenylpropanoid pathway beyond flavonoids, such as lignin or phenolic acid biosynthesis, which could explain its broader metabolic impact. This integrated research approach will enable precise optimization of flavonoid composition in mulberry, facilitating the scalable production of high-value mulberry-derived functional food ingredients.

## 5 Conclusion

We demonstrate that MaMYB12 and MaMYB308 antagonistically regulate flavonoid biosynthesis in mulberry through direct targeting of key biosynthetic genes. This study advances our understanding of transcriptional regulation in woody plants and provides genetic tools for breeding mulberry varieties with enhanced flavonoid content. By enabling the development of high-value flavonoid-rich mulberry products, our findings support the integration of mulberry into sustainable functional food systems, contributing to the growing bioeconomy of natural food ingredients.

## CRediT authorship contribution statement

**Shengmei Han:** Writing – original draft, Project administration. **Teng Zhao:** Writing – original draft, Validation, Investigation. **Jiaqi Zhao:** Investigation, Data curation. **Xiaoyan Liu:** Validation, Investigation. **Jing Xiao:** Investigation, Data curation. **Hong Huang:** Resources, Investigation, Data curation. **Zhaoyang Liu:** Visualization, Project administration. **Yingping Gai:** Writing – review & editing, Supervision, Project administration, Funding acquisition. **Xianling Ji:** Writing – review & editing, Supervision, Funding acquisition.

## Declaration of competing interest

The authors declare that they have no known competing financial interests or personal relationships that could have appeared to influence the work reported in this paper.

## Data Availability

Data will be made available on request.

## Appendix A Supplementary data

Supplementary material: Supplementary Figure S1. Phylogeny of mulberry R2R3-MYB candidates and conserved motif analysis of MaMYB12/MaMYB308. Image 1
