# Supplementary material for: MaMYB12 and MaMYB308 antagonistically regulate flavonoid biosynthesis in mulberry (Morus alba): Implications for functional food ingredient development
Source: Food Chem (Oxf). 2026 Mar 24;12:100395. doi: 10.1016/j.fochms.2026.100395 (PMC13054406; doi:10.1016/j.fochms.2026.100395)
Supplement: Supplementary file 1 — Supplementary material: Supplementary Figure S1. Phylogeny of mulberry R2R3-MYB candidates and conserved motif analysis of MaMYB12/MaMYB308. [file mmc1.docx]

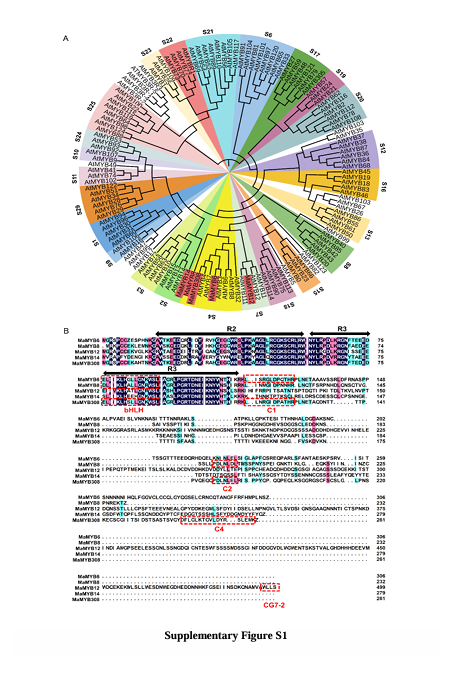


**Supplementary Figure S1. Phylogeny of mulberry R2R3-MYB candidates and conserved motif analysis of MaMYB12/MaMYB308.** (A) Phylogenetic tree of candidate mulberry MYBs and representative *Arabidopsis* R2R3-MYB members from subgroups 4–7. The tree was constructed using the Neighbor-Joining (NJ) method in MEGA 6.0 software (Bootstrap = 1000 replicates). Mulberry candidates are highlighted in red. (B) Conserved motif analysis of MaMYB12 and MaMYB308. Red box indicates the SG7-specific [W/x][L/x]LS conserved motif; blue box marks the SG4-type EAR repression motif (pdLNL[D/E]L), with the core functional domain underlined.
